# Brown Yar Ko Rice Protects Against Hyperglycemia‐Induced Endothelial Injury via Antioxidant and SIRT1 Activation

**DOI:** 10.1155/sci5/6749863

**Published:** 2026-04-06

**Authors:** Witchuda Payuhakrit, Pimchanok Panpinyaporn, Pitchapa Wantanachaisaeng, Teerapat Teeppaibul, Chatsuda Surachaijarin, Chonnapat Naktubtim, Ratsada Praphasawat, Sarawoot Palipoch

**Affiliations:** ^1^ Department of Pathobiology, Faculty of Science, Mahidol University, Bangkok, Thailand, mahidol.ac.th; ^2^ Pathobiology Information and Learning Center, Department of Pathobiology, Faculty of Science, Mahidol University, Bangkok, Thailand, mahidol.ac.th; ^3^ Department of Pathology, School of Medicine, University of Phayao, Phayao, Thailand, up.ac.th; ^4^ School of Medicine, Walailak University, Nakhon Si Thammarat, Thailand, wu.ac.th

**Keywords:** antioxidant, brown Yar Ko rice, endothelial cell injury, hyperglycemia, oxidative stress, sirtuin 1

## Abstract

Endothelial dysfunction and oxidative stress caused by hyperglycemia are the main contributors to vascular complications in patients with diabetes mellitus (DM), which is a significant global health issue. The downregulation of sirtuin 1 (SIRT1), a key regulator of antioxidant defenses, further exacerbates vascular damage in diabetic conditions. This study aimed to investigate the therapeutic effects of brown Yar Ko (YK) rice extract, a traditional variety from southern Thailand, on hyperglycemia‐induced oxidative stress in human umbilical vein endothelial cells (HUVECs), with a focus on SIRT1 activation. Methodologically, YK rice was extracted and identified via triple quadrupole GC‒MS/MS. Antioxidant activity was assessed via DPPH and FRAP assays. A 30 mM glucose‐stimulated HUVEC model was utilized to mimic diabetes in vitro. Oxidative stress, endothelial function, and DNA damage were evaluated via DCFH‐DA, tubulogenesis, and γ‐H2AX staining, respectively. SIRT1 expression was analyzed via western blotting, and downstream signaling pathways were predicted via pharmacological network analysis. The results of chemical profiling revealed that YK rice extract is rich in beneficial fatty acids (linoleic, oleic, and palmitic acids) and micronutrients. The extract presented a high total phenolic content and potent antioxidant activity, significantly reducing reactive oxygen species (ROS) levels and DNA damage in hyperglycemia‐induced HUVECs. Functional assays demonstrated that YK rice extract improved angiogenic capacity and mitigated endothelial dysfunction under hyperglycemic conditions. Mechanistically, treatment with YK rice extract upregulated SIRT1 expression, as confirmed by immunofluorescence and western blot analyses. Network pharmacology revealed the SIRT1 with PPAR‐α and PPAR‐γ axis as a key pathway modulated by major YK rice constituents, potentially suggesting enhanced mitochondrial biogenesis and antioxidant gene expression. However, these proposed mechanisms require further experimental validation for definitive elucidation.

## 1. Introduction

Diabetes mellitus (DM) is a leading noncommunicable disease and a major global health challenge. In 2021, approximately 529 million people worldwide were living with diabetes, and this number is projected to increase substantially in the coming decades [1]. Diabetic vascular complications, including microvascular disorders (retinopathy, nephropathy, cardiomyopathy, and neuropathy) and macrovascular diseases (coronary artery disease, cerebrovascular disease, and peripheral vascular disease), are the principal causes of morbidity and mortality among patients with diabetes [2]. Among the various pathogenic factors, chronic hyperglycemia plays a central role in driving vascular injury, particularly through its deleterious effects on endothelial cells.

Sustained hyperglycemia induces excessive reactive oxygen species (ROS) production, leading to oxidative stress and impaired nitric oxide bioavailability, thereby disrupting endothelial homeostasis. This endothelial dysfunction is a key initiating event in diabetic vasculopathy and contributes to both microvascular and macrovascular complications [3, 4]. Given the limited efficacy and potential adverse effects of current pharmacological interventions, dietary and natural product–based strategies targeting oxidative stress and endothelial dysfunction have attracted increasing attention for diabetes prevention and management.

Sirtuin 1 (SIRT1), a class III histone deacetylase, is a critical regulator of metabolic homeostasis, oxidative stress resistance, inflammation, and aging [5]. Accumulating evidence indicates that SIRT1 exerts vasculoprotective effects in diabetes by attenuating oxidative stress and preserving endothelial function. However, hyperglycemic conditions suppress SIRT1 expression in vascular tissues, thereby aggravating oxidative stress, inflammation, endothelial senescence, and vascular dysfunction [6–10]. Mechanistically, SIRT1 enhances antioxidant defenses through the upregulation of enzymes such as superoxide dismutase 2 (SOD2) [11], promotes mitochondrial biogenesis via PGC‐1α activation [12], and cooperates with AMPK to inhibit ROS‐generating pathways, including NADPH oxidase [13]. Thus, identifying natural SIRT1 activators represents a promising approach to prevent hyperglycemia‐induced endothelial injury.

Rice (Oryza sativa L.) is a staple food consumed globally, and its health impact varies considerably depending on the degree of processing. Brown rice retains the bran and germ layers, providing higher levels of dietary fiber, unsaturated fatty acids, and bioactive phytochemicals compared with white rice. Although pigmented rice varieties have been extensively investigated for their antioxidant and antidiabetic activities, the vasculoprotective efficacy and SIRT1‐modulating effects of traditional Thai brown Yar Ko (YK) rice have not been comparatively evaluated, representing a significant gap in the current literature. While the metabolic benefits of brown rice are increasingly recognized, the biofunctional properties of traditional and region‐specific rice varieties remain underexplored. Brown YK rice, a traditional Thai rice variety cultivated in the Pak Phanang Basin of Nakhon Si Thammarat Province, represents a unique and underinvestigated natural resource with potential health‐promoting properties. Despite its long‐standing cultural significance and local consumption, its protective effects against hyperglycemia‐induced endothelial dysfunction and the underlying molecular mechanisms have not been systematically studied. Therefore, this study aimed to investigate the protective effects of brown YK rice extract against oxidative stress under hyperglycemic conditions and endothelial dysfunction, with a particular focus on SIRT1‐mediated antioxidant and metabolic signaling pathways. By addressing this gap, our findings provide new insight into the potential of a traditional Thai rice variety as a functional food for the prevention of diabetes‐related vascular complications.

## 2. Materials and Methods

### 2.1. Rice Extraction

Brown YK rice obtained from the Rice Research Center, Nakhon Si Thammarat Province, Thailand, was ground into powder (100 g per sample, *n* = 3) and extracted with 90% (v/v) ethanol–water using ultrasonication (90 s, room temperature). The extraction was repeated twice, and the combined extracts were filtered, concentrated by freeze‐drying and air evaporation, stored at −80°C protected from light, and filtered through a membrane prior to analysis.

### 2.2. Phytochemical Identification via Triple Quadrupole GC‒MS/MS

Phytochemical profiling was conducted via a Triple Quadrupole GC‒MS/MS system (GC‐QQQQ) equipped with a static headspace sampler (7890 B GC system and 7000 C GC/MS Triple Quad, Agilent Technologies, Santa Clara, USA). Each sample was dissolved in ethanol, and 1 μL was injected onto an HP‐INNOWAX column (30 m × 0.25 mm, 0.25 μm). Helium served as the carrier gas at a constant flow rate of 1 mL/min. The oven temperature program was as follows: initial temperature of 50°C (held for 2 min), increased to 260°C, and held for 30 min. The EI ion source was operated at 70 eV and 250°C. Mass scanning was performed in full‐scan mode from 33 to 500 amu. Compound identification was based on spectral matches with the NIST 2020 database, with a minimum similarity threshold of 80%.

### 2.3. Nutrition Composition Analysis

Nutritional analysis of the YK rice extract was conducted at the Central Laboratory, Thailand, via standard analytical methods. The parameters analyzed included vitamins (B1, B2, and B3), ash, carbohydrate, fat, moisture, protein, and mineral contents (magnesium, phosphorus, potassium, sodium, and zinc).

### 2.4. Determination of Total Phenolic Compound (TPC) Content

The TPC was determined following the Folin–Denis method. Briefly, 20 μL of YK rice extract or gallic acid standard (Sigma‐Aldrich, Singapore) was mixed with 120 μL of Folin–Denis reagent and 1880 μL of sodium bicarbonate, resulting in a final volume of 2 mL. The mixture was incubated for 30 min at room temperature. The absorbance was measured at 765 nm, and the results are expressed as μg gallic acid equivalents per gram of extract (μg GAE/g) [14].

### 2.5. Cell Culture

Human umbilical vein endothelial cells (HUVECs; ATCC, USA) were cultured in Endothelial Cell Growth Medium‐2 (low serum) supplemented with 1% antibiotic‐antimycotic mixture. The cells were maintained at 37°C in a humidified incubator with 5% CO_2_. For high‐glucose (HG) conditions, cells were treated with 30 mM glucose for 24 h. Cotreated groups received 30 mM glucose plus YK rice extract at either 1 or 10 μg/mL for 24 h [15].

### 2.6. Determination of the Antioxidant Activity of YK Rice Extracts

Antioxidant activity was evaluated using DPPH and ferric reducing antioxidant power (FRAP) assays following established methods [16, 17]. For the DPPH assay, ethanol‐based DPPH solution (2 mM) was calibrated to an absorbance of 0.80 ± 0.05 at 517 nm, reacted with the extract, and antioxidant activity was expressed as mg vitamin C equivalent/g sample. The FRAP assay was performed using acetate buffer (pH 3.6), 2,4,6‐tripyridyl‐s‐triazine, and ferric chloride, with absorbance measured at 593 nm and results expressed as mM FeSO_4_·7H_2_O equivalent/g sample.

### 2.7. Determination of the Cytotoxicity of YK Rice Extract via the MTT Assay

HUVECs were seeded in 96‐well plates and incubated for 24 h. The cells were then treated with various concentrations of YK rice extract for another 24 h. After treatment, MTT solution was added, and the mixture was incubated for 2 h. Then, DMSO was added to dissolve the formazan crystals. The absorbance was measured at 570 nm via a microplate reader [18].

### 2.8. Determination of ROS Levels in HUVECs via DCFH‐DA

HUVECs were seeded in 96‐well plates, incubated for 24 h, and then induced under HG conditions with 30 nM glucose in incomplete M199 media with or without YK rice extract at different concentrations and incubated for 24 h. After treatment, 200 μL of 10 μM DCFH‐DA solution was added to each well and incubated for 1 h. The results were measured at 485/535 nm absorbance with a fluorescence microplate reader [19].

### 2.9. Tubulogenesis Assay

A tubulogenesis assay was performed to investigate the effect of YK rice extract on new blood vessel formation in an in vitro diabetic model. HUVECs (1 × 10^4^ cells/well) were seeded on Matrigel (100 μL/well) in a 96‐well plate under different conditions: HG, HG + YK Low, and HG + YK High for 6 h at 37°C with 5% CO_2_. Medium 199 (M199) nonserum was used as a medium and control for the tubulogenesis assay. Images were captured at 2, 4, and 6 h, and the ability to form new blood vessels was analyzed by the number of junctions and total length via ImageJ software with the angiogenic analyzer program [20].

### 2.10. Immunofluorescence Assay

To study the expression of γ‐H2AX and SIRT‐1 proteins in vascular endothelial cell injury at the genetic level (DNA damage), cells were seeded in 6‐well plates containing coverslips and incubated for 24 h at 37°C in a 5% CO_2_ incubator to allow the cells to adhere to the coverslips. HG conditions were used to simulate diabetic hyperglycemia by adding glucose at a concentration of 30 nM and rice extract at various concentrations in the presence or absence of glucose for 24 h in a cell culture incubator. The cells were stained with a primary antibody (γ‐H2AX or SIRT‐1) and a secondary antibody. Finally, the nuclei were stained with DAPI to study protein expression under a fluorescence microscope [21].

### 2.11. Western Blot Analysis

HUVECs (1 × 10^6^ cells/well) were cultured in 6‐well plates, treated under different conditions for 24 h, and lysed in buffer. The protein concentration was determined via the Bradford assay. Equal amounts of protein (25 μL) were loaded onto SDS‒PAGE gels. After electrophoresis, the proteins were transferred to nitrocellulose membranes, blocked with 5% skim milk, and incubated overnight with primary antibodies against SIRT1. After washing, the membranes were incubated with secondary antibodies (1:1000 dilution) for 1 h. The protein bands were visualized and analyzed via ImageJ software [22].

### 2.12. Target Gene Prediction

The structures of linoleic, oleic, and palmitic acids were obtained from the PubChem database. Their predicted targets were identified via the SwissTargetPrediction, SuperPred, and SEA search servers (species set to *Homo sapiens*). Oxidative stress‐related genes were retrieved from GeneCards, The Human Protein Atlas, and OMIM. Venn diagrams were generated via Venny 2.1 to identify overlapping potential targets of the compounds [22].

### 2.13. Protein Interaction Network

The common genes shared by the compound‐related targets and the oxidative stress‐related targets were obtained via a Venn diagram. The STRING12.0 database (https://cn.string-db.org) was uploaded with the intersecting target data to construct a protein‒protein interaction (PPI) network, and the species was restricted to “*Homo sapiens*.” The PPI network was visualized via Cytoscape 3.10.3 [23], and topology analysis was performed via the CytoHubba plug‐in [24]. The maximal clique centrality (MCC) algorithm was used to identify the top 10 hub proteins among the common proteins.

### 2.14. Statistical Analysis

The data are presented as the means ± standard deviations (SDs) from three independent experiments (*n* = 3). Statistical comparisons were made via one‐way ANOVA in GraphPad Prism version 9. Differences were considered statistically significant at *p* < 0.05.

## 3. Results

### 3.1. Chemical Profiles and Nutritional Composition of YK Rice

**FIGURE 1:**
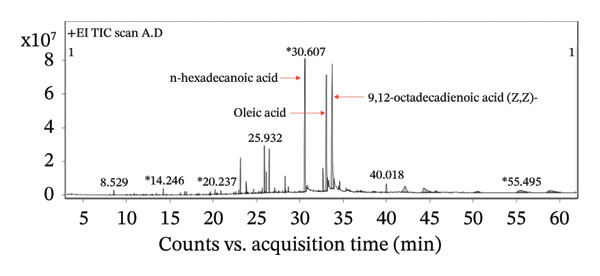
Chromatogram of YK rice via triple quadrupole GC–MS.

The chemical composition of YK rice extract was analyzed via triple quadrupole GC–MS, which identified 40 distinct compounds (Table S1). The three most abundant components were 9,12‐octadecadienoic acid (Z,Z)‐(linoleic acid), oleic acid, and n‐hexadecanoic acid (palmitic acid), as shown in Figure 1 and Table 1. Nutritional analysis further revealed that YK rice extract is rich in vitamin B3, magnesium, phosphorus, and potassium (Table 2). These results suggest that YK rice extract has a favorable fatty acid profile and is enriched with essential micronutrients.

**TABLE 1:** Major chemical composition of YK rice extract.

No.	RT	Name	Molecular formula	MW	Peak area (%)
1	30.607	n‐Hexadecanoic acid	C_16_H_32_O_2_	256	70.43
2	33.082	Oleic Acid	C_18_H_34_O_2_	282	76.24
3	33.764	9,12‐Octadecadienoic acid (Z,Z)‐	C_18_H_32_O_2_	280	100

**TABLE 2:** The nutritional composition of YK rice extract.

Compositions	Unit
Vitamin B1 (Thiamine)	Not detected
Vitamin B2 (Riboflavin)	Not detected
Vitamin B3 (Nicotinic acid)	0.256 mg/100 g
Ash	0.68 g/100 g
Carbohydrate	77.83 g/100 g
Fat	3.42 g/100 g
Moisture	11.02 g/100 g
Protein	7.05 g/100 g
Copper	< 1.00 mg/kg
Iron	5.831 mg/kg
Magnesium	1123.73 mg/kg
Phosphorus	2960.64 mg/kg
Potassium	2360.37 mg/kg
Sodium	< 50.00 mg/kg
Zinc	19.350 mg/kg
Calories	370.30 Kcal/100 g

### 3.2. High Phenolic Content and Antioxidant Activity of YK Rice Extract Attenuate Hyperglycemia‐Induced ROS in HUVECs

To further explore its antioxidant properties, the TPC and antioxidant activity of YK rice extract were evaluated. The extract presented a phenolic content of 9.33 ± 0.06 μg gallic acid equivalent/g sample, indicating notable antioxidant potential (Figures 2(a), 2(d)). DPPH and FRAP assays confirmed this activity, with values of 1.40 ± 0.02 mg vitamin C equivalent/g and 91.13 ± 0.50 mM FeSO_4_·7H_2_O equivalent/g, respectively (Figures 2(b), 2(c), 2(d)).

FIGURE 2The high total phenolic content and antioxidant activity of YK rice extract reduced hyperglycemia‐induced ROS. The absorbance of total phenolic contents of YK rice extract. (a) The percentage of DPPH radical scavenging activity of YK rice extract. (b) The FRAP assay of YK rice extract. (c) The summary of the results: total phenolic content, DPPH, and FRAP after adjusting the measurement value to the standard in the form of an equivalent standard to the sample. (d) The percentage of viable HUVECs for 24 h was determined via the MTT assay. (e) The oxidative stress level in HUVECs under various conditions was determined via DCFH‐DA after 24 h, as shown in 100% of the control. (f) The data are expressed as the means ± SD (*n* = 3); ^∗∗^ *p* < 0.01, and ^∗∗∗∗^ *p* < 0.0001.

**Figure:**
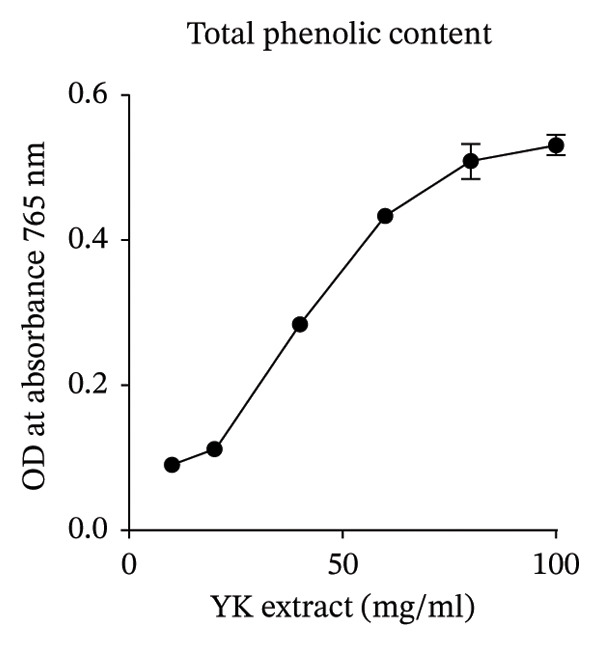
(a)

**Figure:**
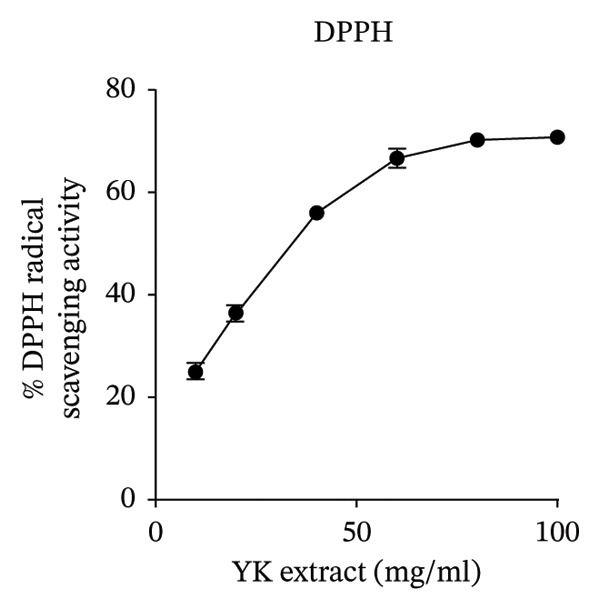
(b)

**Figure:**
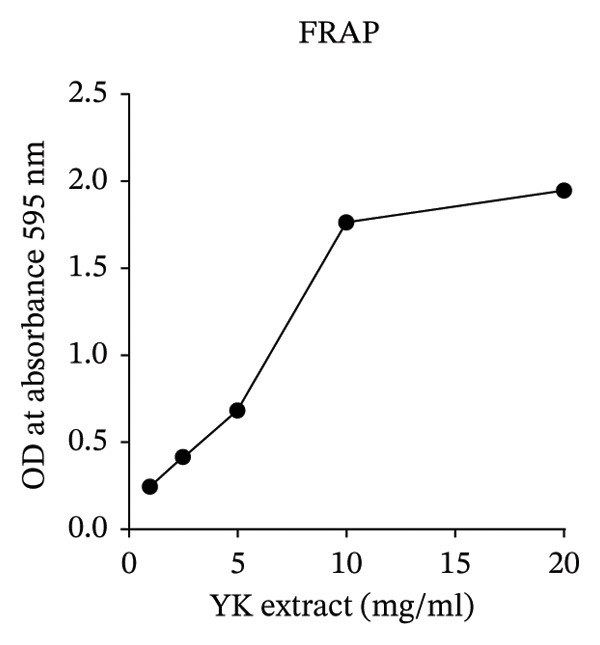
(c)

**Figure:**
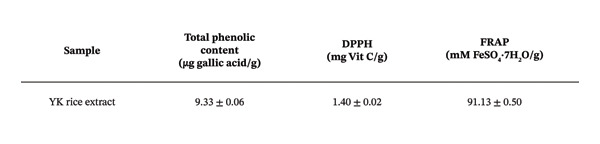
(d)

**Figure:**
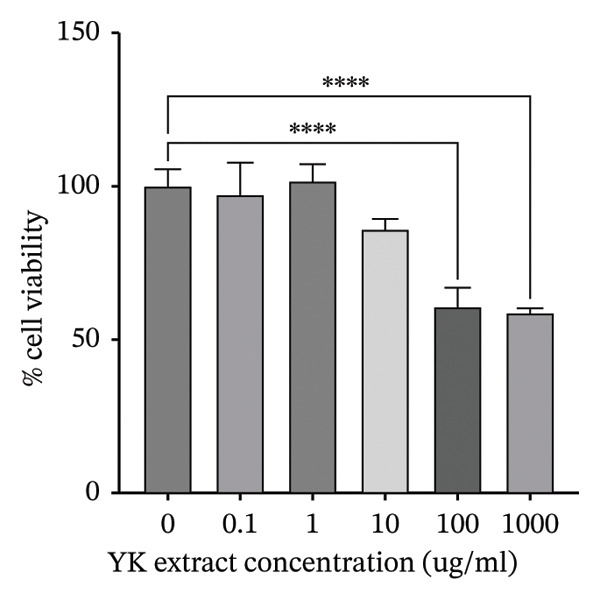
(e)

**Figure:**
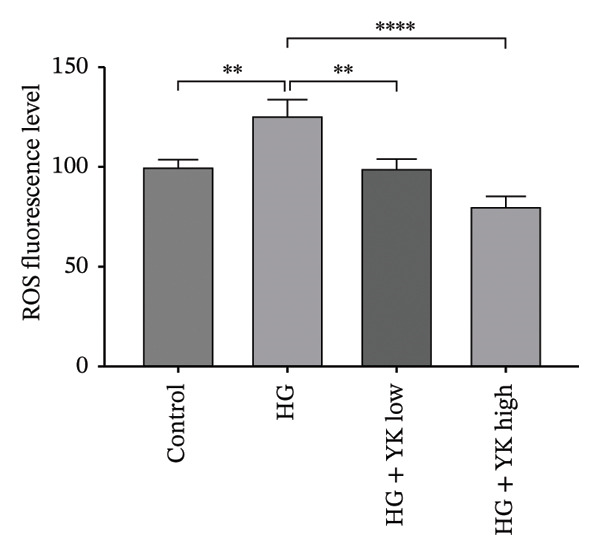
(f)

MTT assays revealed significant cytotoxicity at concentrations of 100 μg/mL (60.84% ± 6.05) and 1000 μg/mL (58.63% ± 1.36), indicating that 1 and 10 μg/mL were used as low and high doses, respectively, for subsequent experiments (Figure 2(e)). Under HG conditions, the ROS level in HUVECs significantly increased to 125.80% ± 7.73 compared with that in the control (100.00% ± 3.52), indicating oxidative stress. Treatment with YK rice extract reduced the ROS level to 90.00% ± 4.90% and 80.00% ± 4.90% at low and high doses, respectively (Figure 2(f)). These findings confirm that YK rice extract exerts potent antioxidant effects and mitigates oxidative stress in hyperglycemic endothelial cells.

### 3.3. YK Rice Extract Inhibits Hyperglycemia‐Induced Endothelial Dysfunction in HUVECs

The effect of YK rice extract on angiogenesis under hyperglycemic conditions was assessed by quantifying the number of junctions and total tube lengths in HUVECs at 2, 4, and 6 h (Figure 3). At 2 h, the numbers of junctions in the control, HG, HG + YK low, and HG + YK high groups were 42 ± 4, 10 ± 9, 41 ± 5, and 50 ± 6, respectively, whereas the tube lengths were 2037 ± 91, 651 ± 100, 1297 ± 53, and 1991 ± 86 μm, respectively. At 4 h, the junctions increased to 60 ± 6, 12 ± 8, 50 ± 4, and 56 ± 6, with tube lengths of 2184 ± 64, 941 ± 91, 1686 ± 42, and 1922 ± 87 μm, respectively. At 6 h, the junctions further increased to 65 ± 2, 17 ± 9, 58 ± 3, and 64 ± 4, with corresponding tube lengths of 2409 ± 69, 1214 ± 55, 1783 ± 39, and 2107 ± 75 μm, respectively. These data show that hyperglycemia impairs angiogenesis, as evidenced by reduced junctions and tube lengths. However, YK rice extract significantly restored angiogenic capacity, indicating its protective effect against endothelial dysfunction under hyperglycemic stress.

FIGURE 3Effects of YK rice extract on new blood vessel formation in HG‐induced HUVECs. Representative images under a 200X microscope at 2, 4 and 6 h, (a) Quantification of the number of junctions, (b) and total length from the tubulogenesis assay under different conditions at 2, 4 and 6 h. (c) Scale bar = 25 μm. The values are expressed as the mean ± standard deviation (SD), ^∗∗∗∗^ *p* < 0.0001.

**Figure:**
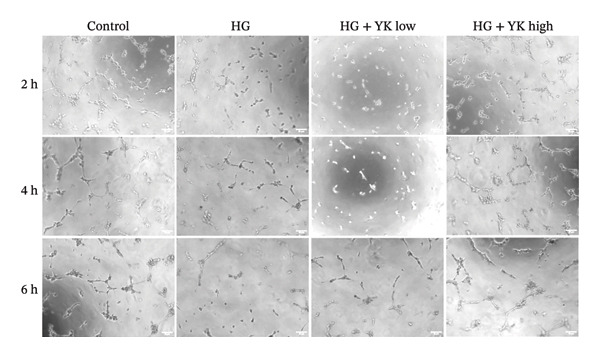
(a)

**Figure:**
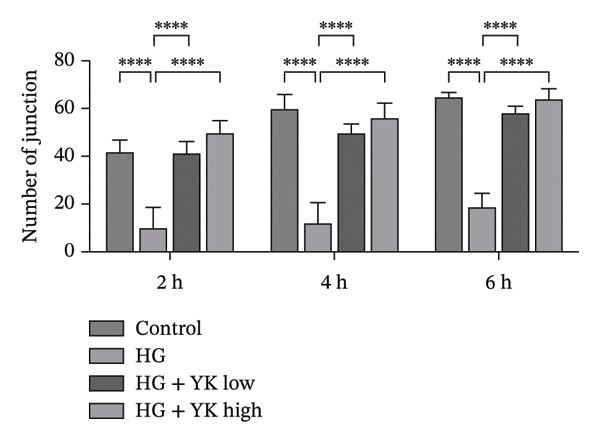
(b)

**Figure:**
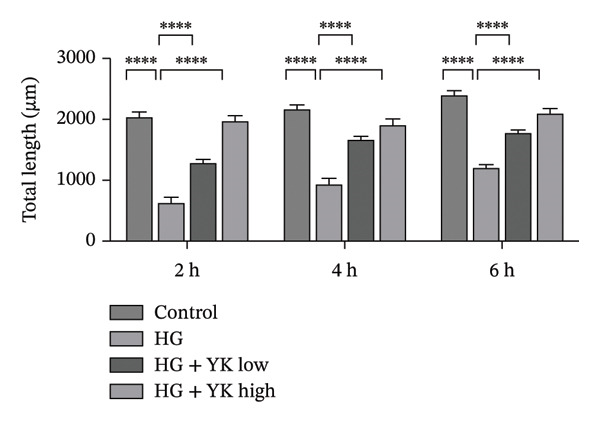
(c)

### 3.4. YK Rice Extract Reduces DNA Damage in Hyperglycemia‐Induced HUVECs

To assess DNA damage, the expression of γ‐H2AX, a marker of DNA double‐strand breaks, was evaluated after 24 h. High‐magnification imaging revealed increased γ‐H2AX foci in HG‐treated cells, whereas no foci were observed in cells treated with YK rice extract (Figure 4(a)). ImageJ analysis revealed that the γ‐H2AX intensities were 3.47 ± 0.58 (control), 13.76 ± 3.13 (HG), 4.63 ± 1.33 (HG + YK low), and 1.52 ± 0.35 (HG + YK high) (Figure 4(b)). Compared with HG treatment, YK rice extract significantly reduced γ‐H2AX expression (*p* < 0.0001), indicating that the extract effectively attenuated DNA damage in hyperglycemia‐exposed endothelial cells.

FIGURE 4Effect of YK rice extract on the inhibition of γ‐H2AX protein expression in hyperglycemia‐induced HUVECs. Expression of the γ‐H2AX protein in hyperglycemic‐induced HUVECs after 24 h of treatment under various conditions under a fluorescence microscope at 1000X magnification, with images cropped to 20 μm × 20 μm. (a) The mean fluorescence intensity of γ‐H2AX was analyzed via ImageJ. (b) Scale bar = 2 μm. The data are presented as the means ± standard deviations (means ± SD) from three independent experiments (*n* = 3). Statistical significance (^∗∗∗∗^ *p* < 0.0001).

**Figure:**
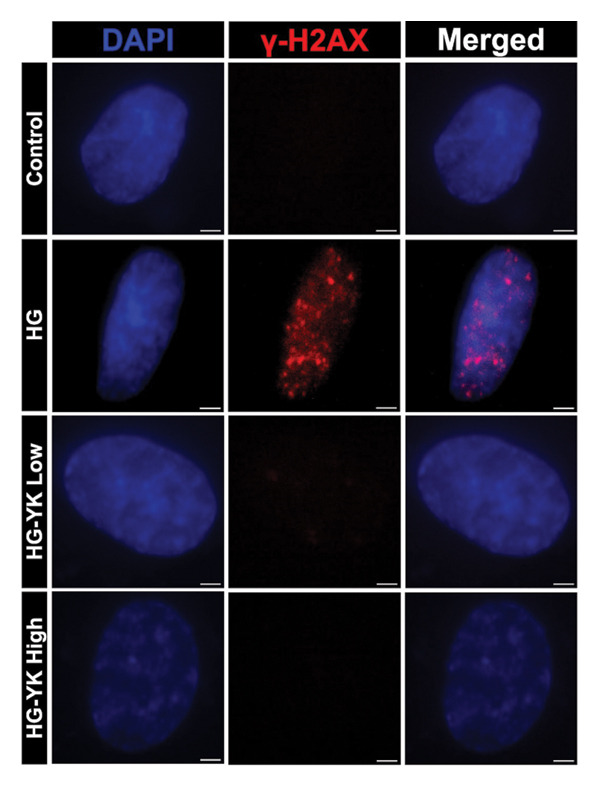
(a)

**Figure:**
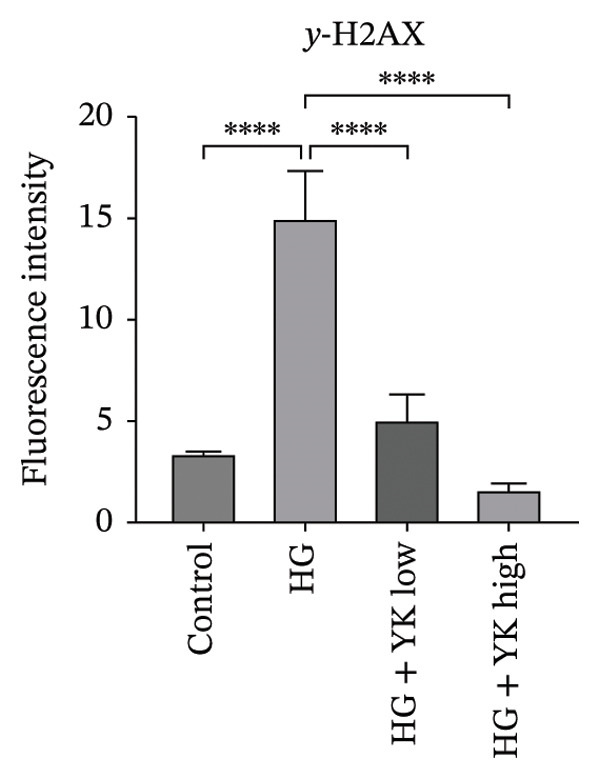
(b)

### 3.5. YK Rice Extract Induced the Expression of SIRT1 in Hyperglycemia‐Induced HUVECs

This study investigated the ability of YK rice extract to attenuate hyperglycemia‐induced ROS generation in HUVECs. Given the critical function of SIRT1 in modulating antioxidant pathways [25], SIRT1 protein expression was quantified via an immunofluorescence assay and western blot analysis to evaluate the ability of YK rice extract to mitigate oxidative stress. After 24 h, the SIRT1 protein expression levels in HUVECs were evaluated at intensities of 7.49 ± 1.39, 5.01 ± 0.81, 6.13 ± 1.20, and 6.90 ± 1.33 in the control, HG, HG with low YK, and HG with high YK groups, respectively. The results indicated that SIRT1 protein expression was significantly greater (*p* < 0.0001) in the group of cells that were treated with both low and high concentrations of YK than in the group of HG‐induced injury endothelial cells, as shown in Figures 5(a), 5(b). In addition, the expression of SIRT1 was confirmed via a western blot analysis, as shown in Figures 5(c), 5(d). Consistent with the results of the fluorescence assay, western blot analysis revealed that SIRT1 expression was lower in the HG condition than in the control, whereas compared with that in the HG condition, the level of SIRT1 expression in the YK rice extract was significantly greater. These results suggest that YK rice extract has the potential to mitigate oxidative stress, potentially via the upregulation of SIRT1 expression in hyperglycemic‐induced endothelial cells.

FIGURE 5YK rice extract induced the expression of SIRT1 in hyperglycemia‐induced HUVECs. SIRT1 protein expression in HUVECs treated with YK rice extract after 24 h, as determined via fluorescence microscopy at 1000X magnification. (a) The bar graphs of the fluorescence intensity of SIRT1 protein expression were analyzed via ImageJ. (b) A representative western blot analysis showing SIRT1 expression; β‐actin was used as the internal control. (c) The expression of SIRT1 is normalized to β‐actin and shown as relative density compared to the control. (d) Scale bar = 5 μm. The data are presented as the means ± standard deviations (means ± SD) from three independent experiments (*n* = 3). Statistical significance (^∗∗∗∗^ *p* < 0.0001).

**Figure:**
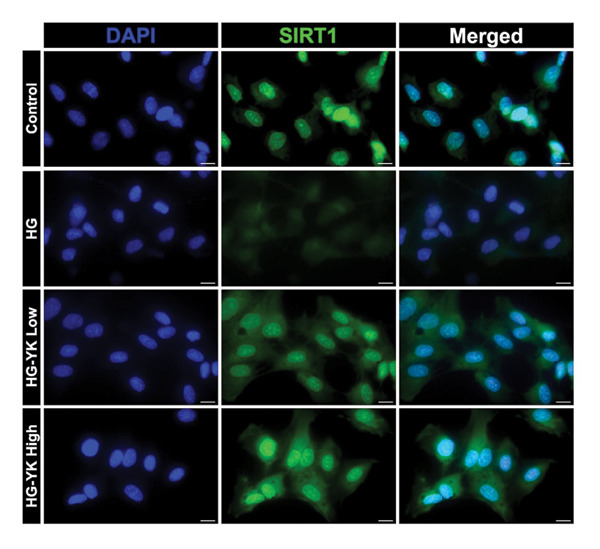
(a)

**Figure:**
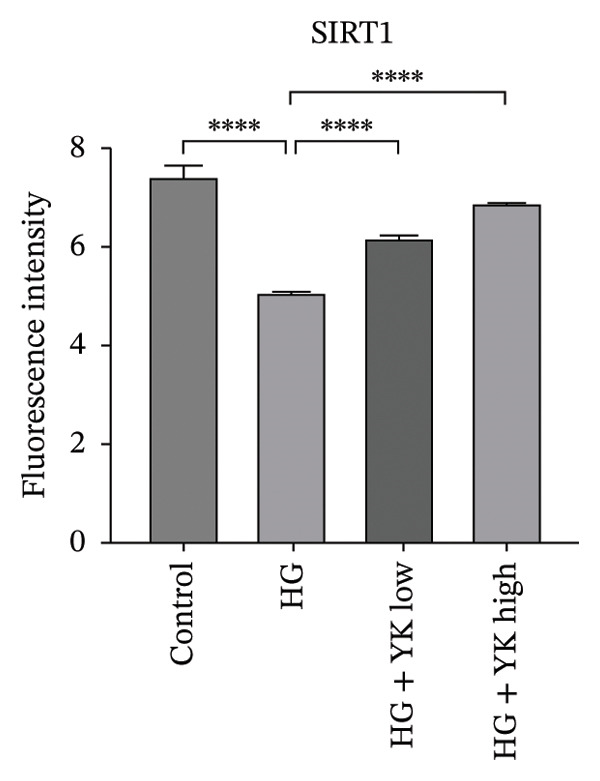
(b)

**Figure:**
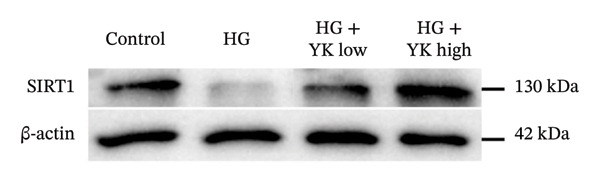
(c)

**Figure:**
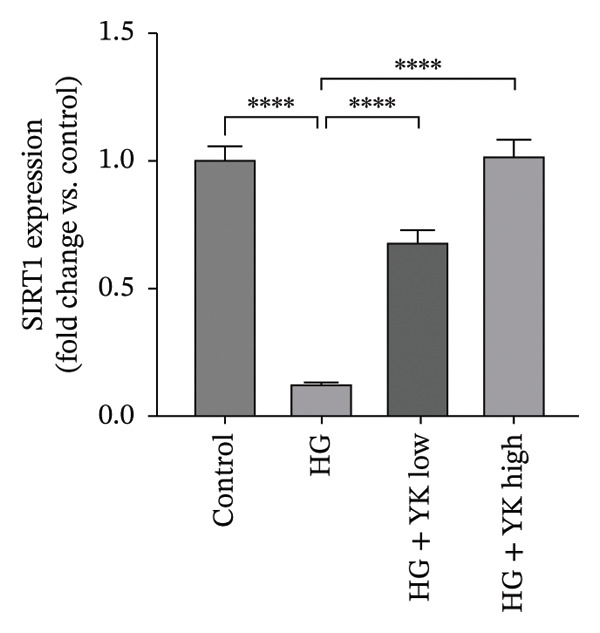
(d)

### 3.6. YK Rice Extract Reduced Endothelial Damage Potentially Through Mitochondrial Biogenesis and Antioxidant Gene Expression

To elucidate the downstream signaling mechanisms by which SIRT1 mitigates oxidative stress‐induced endothelial cell damage, in silico analysis was employed to study SIRT1‐regulated pathways and identify key molecular targets involved in antioxidant defense. Network pharmacology analysis of the major constituents of YK rice extract, including linoleic acid, oleic acid, and palmitic acid, within oxidative stress‐associated pathways revealed 81 shared molecular targets, suggesting their mechanistic role in modulating oxidative damage, as shown in Figure 6(a).

FIGURE 6Network pharmacological analysis of the major compounds in YK rice extract and their associations with SIRT1‐mediated antioxidant activity. Venn diagram showing the intersection between three main compounds in YK rice extract and oxidative stress (a). The protein–protein interaction (PPI) data were generated through STRING and Cytoscape via the maximal clique centrality (MCC) algorithm to illustrate the top 10 hub protein, where each line represents a protein relationship and each node represents a protein. The node color and score are correlated, with darker and more red nodes signifying higher MCC scores (b).

**Figure:**
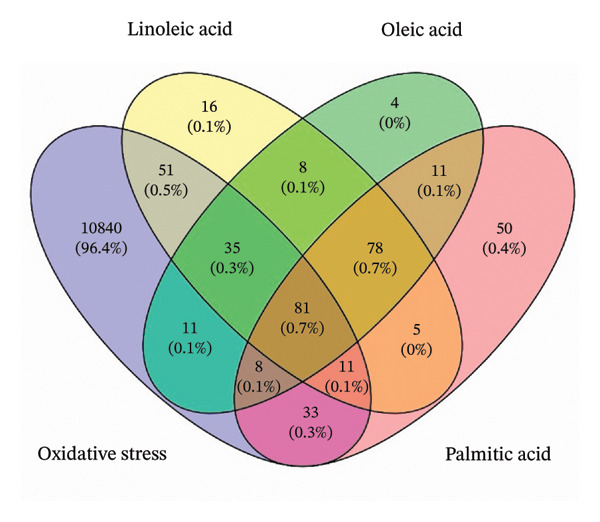
(a)

**Figure:**
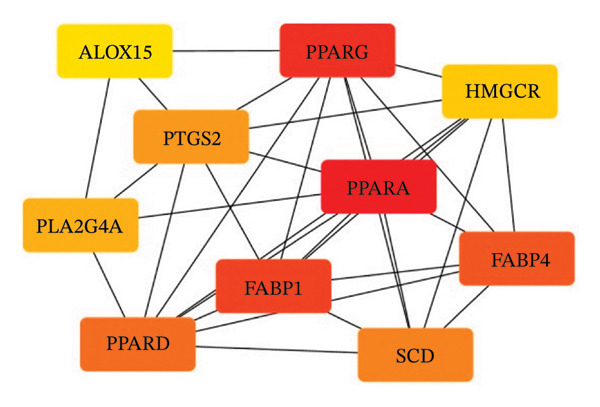
(b)

PPIs were generated through STRING and Cytoscape via the MMC method to illustrate the top 10 targets, where each line represents a protein interaction and each node represents a target. The node color and score are correlated, with darker and more red nodes signifying higher scores (Figure 6(b)). PPAR‐α showed the highest degree (score 2853), and PPAR‐γ ranked second (score 2842), suggesting that they are strongly linked to each other and could be hub targets. These results suggest that major compounds in YK rice extract modulate the SIRT1‐PPAR‐α/γ axis, with PPAR‐α/γ serving as regulatory hubs linking its fatty acids to SIRT1‐mediated antioxidant defense pathways.

## 4. Discussion

Analysis of brown YK rice revealed that the three most prevalent fatty acids were linoleic acid, palmitic acid, and oleic acid. These fatty acids, which are commonly found in plant oils, possess well‐established nutritional and functional properties. Thiamine and riboflavin were not detected in the YK rice extract by GC–MS, which is likely attributable to their intrinsic chemical properties and the limitations of the analytical technique. GC–MS is primarily suited for the analysis of volatile and thermally stable compounds, whereas these water‐soluble B‐vitamins are highly polar, nonvolatile, and thermally labile in their native forms, making them unsuitable for direct GC–MS detection [26, 27]. Unsaturated fatty acids such as linoleic and oleic acid are particularly known for their cardiovascular benefits, including anti‐inflammatory effects and the ability to lower LDL cholesterol levels [28]. Additionally, a previous study demonstrated that α‐linolenic acid can reduce the degree of apoptosis induced by elevated glucose levels in HUVECs [29]. The antioxidant potential of YK rice extract was evaluated, revealing high antioxidant activity. However, a discrepancy was observed between different assays: the DPPH assay showed relatively low antioxidant activity, while both the TPC and the FRAP assays indicated significant antioxidant capacity. This inconsistency may be attributed to the hydrophilic nature of the extract, which tends to be less reactive in the DPPH assay [30]. However, additional assays, such as ABTS and ORAC, would provide further support and improve the consistency of the antioxidant evaluation. Moreover, YK rice showed potent antioxidant activity and exhibited comparable efficacy to rice varieties reported in previous studies, such as pigmented rice [31] and Sangyod rice [32].

The tubulogenesis assay revealed that HG concentrations impair HUVEC function, disrupting their ability to form organized vascular networks. These findings are consistent with previous research demonstrating that hyperglycemia induces endothelial apoptosis [33], elevates oxidative stress [34], and promotes inflammation and ROS generation, all of which contribute to vascular damage and impaired angiogenesis under diabetic conditions [35–37]. Importantly, treatment with YK rice extract under HG conditions significantly improved angiogenesis, as indicated by increased junction formation and total tube length, suggesting a protective effect and the ability to support angiogenic activity in HUVECs under diabetic‐like stress.

Given that hyperglycemia induces endothelial dysfunction, the underlying molecular mechanisms were investigated. Treatment with YK rice extract significantly reduced the expression of γ‐H2AX, a biomarker of DNA damage, in HUVECs exposed to HG. These findings suggest that the extract has the capacity to mitigate DNA damage in endothelial cells. Previous studies have shown that HG levels induce γ‐H2AX expression in the endothelial progenitor cells of diabetic mice, indicating glucose‐mediated genotoxic stress [38]. Furthermore, SIRT1 expression was evaluated. HG conditions led to a marked decrease in SIRT1 levels in HUVECs. However, treatment with YK rice extract restored SIRT1 expression in a dose‐dependent manner, suggesting that the extract may counteract the hyperglycemia‐induced suppression of SIRT1. This finding is consistent with earlier findings that HG reduces SIRT1 expression, leading to oxidative damage, endothelial senescence, and vascular dysfunction [39]. SIRT1 plays a crucial role in endothelial protection by regulating endothelial nitric oxide synthase (eNOS) and promoting endothelial migration and angiogenesis while preventing premature senescence. These findings support the hypothesis that YK rice extract contains bioactive compounds capable of activating SIRT1‐dependent pathways, thereby protecting endothelial cells against HG‐induced oxidative stress and dysfunction.

In silico pathway analysis of SIRT1 downstream signaling identified PPAR‐α and PPAR‐γ as key nodes with the highest degree of interaction among the three major YK rice compounds and oxidative stress‐related pathways, suggesting a functional connection with SIRT1 activity. Previous studies have demonstrated that SIRT1 can deacetylate PGC‐1α, increasing its ability to coactivate PPAR‐α and PPAR‐γ, which in turn promotes mitochondrial biogenesis and the expression of antioxidant genes such as SOD2. [11, 40, 41]. Therefore, PPAR‐α and PPAR‐γ represent potential therapeutic targets influenced by YK rice extract, offering a mechanistic explanation for how these bioactive compounds may modulate SIRT1 signaling and reduce the oxidative stress associated with hyperglycemia. However, this study has several limitations. The findings are based solely on in vitro experiments, with a limited number of tested concentrations, which restricts comprehensive dose–response characterization. Additionally, the absence of positive (e.g., N‐acetylcysteine) and osmotic (e.g., mannitol) controls may limit the interpretation of the ROS, γ‐H2AX, and SIRT1 results, underscoring the need for their inclusion in future studies to strengthen mechanistic rigor. Furthermore, functional validation of PPAR‐α/γ activity, mitochondrial biogenesis, and downstream antioxidant targets (e.g., SOD2) has not yet been performed. Notably, the proposed mechanisms are largely based on network pharmacology predictions and therefore require comprehensive experimental confirmation.

## 5. Conclusions

This study demonstrates that brown YK rice extract, rich in phenolic compounds and beneficial fatty acids, protects endothelial cells from oxidative stress under hyperglycemic conditions, DNA damage, and angiogenic dysfunction by reducing ROS levels and activating the SIRT, potentially via the PPAR‐α/γ signaling pathway involved in mitochondrial biogenesis and antioxidant defense. These findings highlight the therapeutic potential of YK rice extract in protecting vascular endothelial cells from hyperglycemia‐induced damage by targeting oxidative stress pathways and enhancing endothelial resilience.

## Author Contributions

Witchuda Payuhakrit: conceptualization, data curation, formal analysis, investigation, methodology, resources, software, validation, visualization, and writing–original draft. Pimchanok Panpinyaporn: investigation and methodology. Pitchapa Wantanachaisaeng: investigation and methodology. Teerapat Teeppaibul: investigation and methodology. Chatsuda Surachaijarin: investigation and methodology. Chonnapat Naktubtim: investigation and methodology. Ratsada Praphasawat: investigation and methodology. Sarawoot Palipoch: conceptualization, data curation, formal analysis, funding acquisition, investigation, methodology, visualization, and writing–review and editing.

## Funding

This research was financially supported by the Office of the Royal Development Projects Board, Thailand.

## Disclosure

All authors approved the final version of the manuscript to be published.

## Conflicts of Interest

The authors declare no conflicts of interest.

## Supporting Information

Table S1. Chemical composition of YK rice extract.

## Supporting information


**Supporting Information** Additional supporting information can be found online in the Supporting Information section.

## Data Availability

The data that support the findings of this study are available from the corresponding author upon reasonable request.

## References

[1] Ong K. L. , Stafford L. K. , McLaughlin S. A. et al., Global, Regional, and National Burden of Diabetes From 1990 to 2021, With Projections of Prevalence to 2050: A Systematic Analysis for the Global Burden of Disease Study 2021, The Lancet. (July 2023) 402, no. 10397, 203–234, 10.1016/S0140-6736(23)01301-6. PMC10364581 37356446

[2] Yang D.-R. , Wang M.-Y. , Zhang C.-L. , and Wang Y. , Endothelial Dysfunction in Vascular Complications of Diabetes: A Comprehensive Review of Mechanisms and Implications, Frontiers in Endocrinology. (April 2024) 15, 10.3389/fendo.2024.1359255. PMC11026568 38645427

[3] Bhatti J. S. , Sehrawat A. , Mishra J. et al., Oxidative Stress in the Pathophysiology of Type 2 Diabetes and Related Complications: Current Therapeutics Strategies and Future Perspectives, Free Radical Biology and Medicine. (May 2022) 184, 114–134, 10.1016/j.freeradbiomed.2022.03.019. 35398495

[4] An Y. , Xu B. , Wan S. et al., The Role of Oxidative Stress in Diabetes Mellitus-Induced Vascular Endothelial Dysfunction, Cardiovascular Diabetology. (September 2023) 22, no. 1, 10.1186/s12933-023-01965-7. PMC10475205 37660030

[5] Lu C. , Zhao H. , Liu Y. et al., Novel Role of the SIRT1 in Endocrine and Metabolic Diseases, International Journal of Biological Sciences. (2023) 19, no. 2, 484–501, 10.7150/ijbs.78654. 36632457 PMC9830516

[6] Karbasforooshan H. and Karimi G. , The Role of SIRT1 in Diabetic Retinopathy, Biomedicine and Pharmacotherapy. (January 2018) 97, 190–194, 10.1016/j.biopha.2017.10.075, 2-s2.0-85032298949. 29091865

[7] Kume S. , Uzu T. , Kashiwagi A. , and Koya D. , SIRT1, a Calorie Restriction Mimetic, in a New Therapeutic Approach for Type 2 Diabetes Mellitus and Diabetic Vascular Complications, Endocrine, Metabolic and Immune Disorders—Drug Targets. (March 2010) 10, no. 1, 16–24, 10.2174/187153010790827957, 2-s2.0-77950834672. 20044906

[8] Mishra M. , Duraisamy A. J. , and Kowluru R. A. , Sirt1: A Guardian of the Development of Diabetic Retinopathy, Diabetes. (April 2018) 67, no. 4, 745–754, 10.2337/db17-0996, 2-s2.0-85044482709. 29311218 PMC5860853

[9] Orimo M. , Minamino T. , Miyauchi H. et al., Protective Role of SIRT1 in Diabetic Vascular Dysfunction, Arteriosclerosis, Thrombosis, and Vascular Biology. (June 2009) 29, no. 6, 889–894, 10.1161/ATVBAHA.109.185694, 2-s2.0-66349101373. 19286634

[10] Ota H. , Eto M. , Kano M. R. et al., Induction of Endothelial Nitric Oxide Synthase, SIRT1, and Catalase by Statins Inhibits Endothelial Senescence Through the Akt Pathway, Arteriosclerosis, Thrombosis, and Vascular Biology. (November 2010) 30, no. 11, 2205–2211, 10.1161/ATVBAHA.110.210500, 2-s2.0-78149285838. 20705918

[11] Meng T. , Qin W. , and Liu B. , SIRT1 Antagonizes Oxidative Stress in Diabetic Vascular Complication, Frontiers in Endocrinology. (November 2020) 11, 10.3389/fendo.2020.568861. PMC7701141 33304318

[12] Khan R. S. , Fonseca-Kelly Z. , Callinan C. , Zuo L. , Sachdeva M. M. , and Shindler K. S. , SIRT1 Activating Compounds Reduce Oxidative Stress and Prevent Cell Death in Neuronal Cells, Frontiers in Cellular Neuroscience. (2012) 6, 10.3389/fncel.2012.00063, 2-s2.0-84898563412. PMC3533205 23293585

[13] Zarzuelo M. J. , López-Sepúlveda R. , Sánchez M. et al., SIRT1 Inhibits NADPH Oxidase Activation and Protects Endothelial Function in the Rat Aorta: Implications for Vascular Aging, Biochemical Pharmacology. (May 2013) 85, no. 9, 1288–1296, 10.1016/j.bcp.2013.02.015, 2-s2.0-84875727264. 23422569

[14] Im K. H. , Baek S. A. , Choi J. , and Lee T. S. , Antioxidant, Anti-Melanogenic and Anti-Wrinkle Effects of *Phellinus vaninii* , Mycobiology. (October 2019) 47, no. 4, 494–505, 10.1080/12298093.2019.1673595. 32010471 PMC6968557

[15] Han X. , Wang B. , Sun Y. et al., Metformin Modulates High Glucose-Incubated Human Umbilical Vein Endothelial Cells Proliferation and Apoptosis Through AMPK/CREB/BDNF Pathway, Frontiers in Pharmacology. (November 2018) 9, 10.3389/fphar.2018.01266, 2-s2.0-85056266665. PMC6232387 30459620

[16] Brand-Williams W. , Cuvelier M. E. , and Berset C. , Use of a Free Radical Method to Evaluate Antioxidant Activity, LWT--Food Science and Technology. (1995) 28, no. 1, 25–30, 10.1016/S0023-6438(95)80008-5, 2-s2.0-58149364663.

[17] Benzie I. F. F. and Strain J. J. , The Ferric Reducing Ability of Plasma (FRAP) as a Measure of ‘Antioxidant Power’: The FRAP Assay, Analytical Biochemistry. (July 1996) 239, no. 1, 70–76, 10.1006/abio.1996.0292, 2-s2.0-0030586361. 8660627

[18] Payuhakrit W. , Panpinyaporn P. , Khumsri W. et al., Enhancing Chronic Wound Healing With Thai Indigenous Rice Variety, Kaab Dum: Exploring ER Stress and Senescence Inhibition in HaCaT Keratinocyte Cell Line, PLoS One. (May 2024) 19, no. 5, 10.1371/journal.pone.0302662. PMC11095683 38748716

[19] Suwannalert P. , Panpinyaporn P. , Wantanachaisaeng P. et al., 17-AAG Induces Endoplasmic Reticulum Stress-Mediated Apoptosis in Breast Cancer Cells, Possibly Through PERK/eIF2α Up-regulation, In Vivo. (2024) 38, no. 5, 2228–2238, 10.21873/invivo.13687. 39187325 PMC11363749

[20] Uttarawichien T. , Kamnerdnond C. , Inwisai T. , Suwannalert P. , Sibmooh N. , and Payuhakrit W. , Quercetin Inhibits Colorectal Cancer Cells Induced-Angiogenesis in Both Colorectal Cancer Cell and Endothelial Cell Through Downregulation of VEGF-A/VEGFR2, Scientia Pharmaceutica. (May 2021) 89, no. 2, 10.3390/scipharm89020023.

[21] Nwe S. Y. , Uttarawichien T. , Boonsom T. et al., Bioassay-Guided Isolation of Two Antiproliferative Metabolites From Pterocarpus Indicus Willd. Against TGF-β-Induced Prostate Stromal Cells (WPMY-1) Proliferation via PI3K/AKT Signaling Pathway, Frontiers in Pharmacology. (October 2024) 15, 10.3389/fphar.2024.1452887. PMC11483373 39421674

[22] Tanomrat R. , Payuhakrit W. , Naktubtim C. et al., Pharmacological and in Vitro Studies on Quercetin-Rich Onion Peel Extract (*Allium cepa*) and N-Acetylcysteine Enhancing Apoptosis in Colorectal Cancer, Trends in Sciences. (January 2025) 22, no. 3, 10.48048/tis.2025.9111.

[23] Szklarczyk D. , Kirsch R. , Koutrouli M. et al., The STRING Database in 2023: Protein–Protein Association Networks and Functional Enrichment Analyses for Any Sequenced Genome of Interest, Nucleic Acids Research. (January 2023) 51, no. D1, D638–D646, 10.1093/nar/gkac1000. 36370105 PMC9825434

[24] Chin C.-H. , Chen S.-H. , Wu H.-H. , Ho C.-W. , Ko M.-T. , and Lin C.-Y. , Cytohubba: Identifying Hub Objects and Sub-Networks From Complex Interactome, BMC Systems Biology. (December 2014) 8, no. S4, 10.1186/1752-0509-8-S4-S11, 2-s2.0-84961596645. PMC4290687 25521941

[25] Singh C. K. , Chhabra G. , Ndiaye M. A. , Garcia-Peterson L. M. , Mack N. J. , and Ahmad N. , The Role of Sirtuins in Antioxidant and Redox Signaling, Antioxidants and Redox Signaling. (March 2018) 28, no. 8, 643–661, 10.1089/ars.2017.7290, 2-s2.0-85042195920. 28891317 PMC5824489

[26] Tang X. , Cronin D. A. , and Brunton N. P. , A Simplified Approach to the Determination of Thiamine and Riboflavin in Meats Using Reverse Phase HPLC, Journal of Food Composition and Analysis. (December 2006) 19, no. 8, 831–837, 10.1016/j.jfca.2005.12.013, 2-s2.0-33747800293.

[27] Porter K. and Lodge J. K. , Determination of Selected Water-Soluble Vitamins (Thiamine, Riboflavin, Nicotinamide and Pyridoxine) From a Food Matrix Using Hydrophilic Interaction Liquid Chromatography Coupled With Mass Spectroscopy, Journal of Chromatography B. (May 2021) 1171, 10.1016/j.jchromb.2021.122541. 33773258

[28] Wang Q. , Zhang H. , Jin Q. , and Wang X. , Effects of Dietary Linoleic Acid on Blood Lipid Profiles: A Systematic Review and Meta-Analysis of 40 Randomized Controlled Trials, Foods. (May 2023) 12, no. 11, 10.3390/foods12112129. PMC10253160 37297374

[29] Zhang W. , Wang R. , Han S. et al., α-Linolenic Acid Attenuates High Glucose-Induced Apoptosis in Cultured Human Umbilical Vein Endothelial Cells via PI3K/Akt/eNOS Pathway, Nutrition. (October 2007) 23, no. 10, 762–770, 10.1016/j.nut.2007.07.003, 2-s2.0-34548611664. 17716867

[30] Rumpf J. , Burger R. , and Schulze M. , Statistical Evaluation of DPPH, ABTS, FRAP, and Folin-Ciocalteu Assays to Assess the Antioxidant Capacity of Lignins, International Journal of Biological Macromolecules. (April 2023) 233, 10.1016/j.ijbiomac.2023.123470. 36736974

[31] Callcott E. T. , Blanchard C. L. , Oli P. , and Santhakumar A. B. , Pigmented Rice‐Derived Phenolic Compounds Reduce Biomarkers of Oxidative Stress and Inflammation in Human Umbilical Vein Endothelial Cells, Molecular Nutrition and Food Research. (December 2018) 62, no. 24, 10.1002/mnfr.201800840, 2-s2.0-85056830870. 30382609

[32] Woonnoi W. , Thipart K. , Hanchang W. et al., Sangyod Rice Extract Attenuates Vascular Inflammation and Injury in a Rat Model of Diabetes by Modulating the Akt/MAPK Signaling Pathway, Advances in Pharmacological and Pharmaceutical Sciences. (January 2025) 2025, no. 1, 10.1155/adpp/1169062. PMC12021485 40276785

[33] Liu L. , Huang S. , Xu M. et al., Isoquercitrin Protects HUVECs Against High Glucose-Induced Apoptosis Through Regulating p53 Proteasomal Degradation, International Journal of Molecular Medicine. (May 2021) 48, no. 1, 10.3892/ijmm.2021.4955. PMC8121554 33982778

[34] Uruski P. , Mikuła-Pietrasik J. , Drzewiecki M. et al., Diverse Functional Responses to High Glucose by Primary and Permanent Hybrid Endothelial Cells in Vitro, Journal of Molecular and Cellular Cardiology. (July 2021) 156, 1–6, 10.1016/j.yjmcc.2021.03.004. 33731316

[35] Alves-Lopes R. , Neves K. B. , Montezano A. C. et al., Internal Pudental Artery Dysfunction in Diabetes Mellitus is Mediated by NOX1-Derived ROS-Nrf2-and Rho Kinase–Dependent Mechanisms, Hypertension. (October 2016) 68, no. 4, 1056–1064, 10.1161/HYPERTENSIONAHA.116.07518, 2-s2.0-84981703929. 27528061

[36] Pal P. B. , Sonowal H. , Shukla K. , Srivastava S. K. , and Ramana K. V. , Aldose Reductase Regulates hyperglycemia-Induced HUVEC Death via SIRT1/AMPK-α1/mTOR Pathway, Journal of Molecular Endocrinology. (July 2019) 63, no. 1, 11–25, 10.1530/JME-19-0080, 2-s2.0-85067174763. 30986766 PMC6555667

[37] Shi Y. and Vanhoutte P. M. , Macro‐ and Microvascular Endothelial Dysfunction in Diabetes: Diabetes-Induced Endothelial Cell Dysfunction, Journal of Diabetes. (May 2017) 9, no. 5, 434–449, 10.1111/1753-0407.12521, 2-s2.0-85014010148. 28044409

[38] Heng Y.-Y. , Shang H.-J. , Zhang X. , and Wei W. , Sodium Tanshinone IIA Sulfonate Ameliorates Neointima by Protecting Endothelial Progenitor Cells in Diabetic Mice, BMC Cardiovascular Disorders. (September 2023) 23, no. 1, 10.1186/s12872-023-03485-4. PMC10494373 37697234

[39] Han Y. and Kim S. Y. , Endothelial Senescence in Vascular Diseases: Current Understanding and Future Opportunities in Senotherapeutics, Experimental and Molecular Medicine. (January 2023) 55, no. 1, 1–12, 10.1038/s12276-022-00906-w. 36599934 PMC9898542

[40] Porcuna J. , Mínguez-Martínez J. , and Ricote M. , The PPARα and PPARγ Epigenetic Landscape in Cancer and Immune and Metabolic Disorders, International Journal of Molecular Sciences. (September 2021) 22, no. 19, 10.3390/ijms221910573. PMC8508752 34638914

[41] Kosgei V. J. , Coelho D. , Guéant-Rodriguez R.-M. , and Guéant J.-L. , Sirt1-PPARS Cross-Talk in Complex Metabolic Diseases and Inherited Disorders of the One Carbon Metabolism, Cells. (August 2020) 9, no. 8, 10.3390/cells9081882. PMC7465293 32796716

