# Supplementary material for: Brown Yar Ko Rice Protects Against Hyperglycemia‐Induced Endothelial Injury via Antioxidant and SIRT1 Activation
Source: Scientifica (Cairo). 2026 Apr 6;2026:6749863. doi: 10.1155/sci5/6749863 (PMC13053651; doi:10.1155/sci5/6749863)
Supplement: Supplementary file 1 — Supporting Information Additional supporting information can be found online in the Supporting Information section. [file SCI5-2026-6749863-s001.docx]

**Table 1**: Chemical composition of YK rice extract.

| **No.** | **RT** | **Name** | **Molecular formula** | **MW** | **Peak area**  **(%)** |
| --- | --- | --- | --- | --- | --- |
| 1 | 3.641 | Toluene | C_7_H_8_ | 92 | 0.18 |
| 2 | 8.529 | 2-Heptenal, (E)- | C_7_H_12_O | 112 | 1.05 |
| 3 | 10.585 | Octanoic acid, ethyl ester | C_10_H_20_O_2_ | 172 | 0.05 |
| 4 | 10.875 | Acetic acid | C_2_H_4_O_2_ | 60 | 0.42 |
| 5 | 12.261 | 2,4-Dihydroxy-2,5-dimethyl-3(2H)-furan-3-one | C_6_H_8_O_4_ | 144 | 0.3 |
| 6 | 12.965 | Dimethyl Sulfoxide | C_2_H_6_OS | 78 | 0.25 |
| 7 | 13.462 | Hexadecane | C_16_H_34_ | 226 | 0.33 |
| 8 | 13.718 | 2-Decenal, (Z)- | C_10_H_18_O | 154 | 0.13 |
| 9 | 14.246 | 2-Decenal, (E)- | C_10_H_18_O | 154 | 1.49 |
| 10 | 14.539 | 3-Furanmethanol | C_5_H_6_O_2_ | 98 | 0.12 |
| 11 | 15.127 | Heptadecane | C_17_H_36_ | 240 | 0.11 |
| 12 | 16.226 | 2,4-Decadienal, (E,Z)- | C_10_H_16_O | 152 | 0.61 |
| 13 | 16.716 | Octadecane | C_18_H_38_ | 254 | 0.74 |
| 14 | 16.931 | 2,4-Decadienal, (E,E)- | C_10_H_16_O | 152 | 0.77 |
| 15 | 18.23 | Nonadecane | C_19_H_40_ | 268 | 0.16 |
| 16 | 18.316 | Dimethyl sulfone | C_2_H_6_O_2_S | 94 | 0.48 |
| 17 | 19.687 | Eicosane | C_20_H_42_ | 282 | 1.14 |
| 18 | 20.237 | Isopropyl myristate | C_17_H_34_O_2_ | 270 | 1.29 |
| 19 | 20.399 | Tetradecanoic acid, ethyl ester | C_16_H_32_O_2_ | 256 | 0.47 |
| 20 | 20.501 | Octanoic acid | C_8_H_16_O_2_ | 144 | 0.37 |
| 21 | 22.659 | Hexadecanoic acid, methyl ester | C_17_H_34_O_2_ | 270 | 0.58 |
| 22 | 22.968 | Isopropyl palmitate | C_19_H_38_O_2_ | 298 | 1.01 |
| 23 | 23.156 | Hexadecanoic acid, ethyl ester | C_18_H_36_O_2_ | 284 | 9.91 |
| 24 | 23.838 | Glycerin | C_3_H_8_O_3_ | 92 | 3.44 |
| 25 | 24.674 | 1-Hexadecanol | C_16_H_34_O | 242 | 1.04 |
| 26 | 25.507 | 9-Octadecenoic acid, methyl ester, (E)- | C_19_H_36_O_2_ | 296 | 0.5 |
| 27 | 25.597 | 1-Decanol, 2-hexyl- | C_16_H_34_O | 298 | 0.27 |
| 28 | 25.687 | Octadecanoic acid, ethyl ester | C_20_H_40_O_2_ | 312 | 1.42 |
| 29 | 25.932 | (E)-9-Octadecenoic acid ethyl ester | C_20_H_38_O_2_ | 310 | 14.03 |
| 30 | 26.019 | (E)-9-Octadecenoic acid ethyl ester | C_20_H_38_O_2_ | 310 | 0.49 |
| 31 | 26.154 | 5-Hydroxymethylfurfural | C_6_H_6_O_3_ | 126 | 5.84 |
| 32 | 26.497 | Linoleic acid ethyl ester | C_20_H_36_O_2_ | 308 | 12.79 |
| 33 | 27.104 | 1-Octadecanol | C_18_H_38_O | 270 | 1.17 |
| 34 | 28.309 | Tetradecanoic acid | C_14_H_28_O_2_ | 228 | 4.34 |
| 35 | 30.607 | n-Hexadecanoic acid | C_16_H_32_O_2_ | 256 | 70.43 |
| 36 | 32.687 | Octadecanoic acid | C_18_H_36_O_2_ | 284 | 9.97 |
| 37 | 33.082 | Oleic Acid | C_18_H_34_O_2_ | 282 | 76.24 |
| 38 | 33.764 | 9,12-Octadecadienoic acid (Z,Z)- | C_18_H_32_O_2_ | 280 | 100 |
| 39 | 34.608 | 9,12,15-Octadecatrienoic acid, (Z,Z,Z)- | C_18_H_30_O_2_ | 278 | 3.11 |
| 40 | 44.321 | Hexadecanoic acid, 2-hydroxy-1-(hydroxymethyl)ethyl ester | C_19_H_38_O_4_ | 330 | 16.36 |
